# Comprehensive identification and characterization of simple sequence repeats based on the whole-genome sequences of 14 forest and fruit trees

**DOI:** 10.48130/FR-2021-0007

**Published:** 2021-04-21

**Authors:** Xiaoming Song, Nan Li, Yuanyuan Guo, Yun Bai, Tong Wu, Tong Yu, Shuyan Feng, Yu Zhang, Zhiyuan Wang, Zhuo Liu, Hao Lin

**Affiliations:** 1 School of Life Science and Technology and Center for Informational Biology, University of Electronic Science and Technology of China, Chengdu 610054, China; 2 School of Life Sciences/School of Economics, North China University of Science and Technology, Tangshan, Hebei 063210, China; 3 Food Science and Technology Department, University of Nebraska-Lincoln, Lincoln, NE 68588, USA

**Keywords:** Simple sequence repeat, Comparative analysis, Functional enrichment analysis, Trihelix gene family, AP2 gene family

## Abstract

Simple sequence repeats (SSRs) are popular and important molecular markers that exist widely in plants. Here, we conducted a comprehensive identification and comparative analysis of SSRs in 14 tree species. A total of 16, 298 SSRs were identified from 429, 449 genes, and primers were successfully designed for 99.44% of the identified SSRs. Our analysis indicated that tri-nucleotide SSRs were the most abundant, with an average of ~834 per species. Functional enrichment analysis by combining SSR-containing genes in all species, revealed 50 significantly enriched terms, with most belonging to transcription factor families associated with plant development and abiotic stresses such as Myeloblastosis_DNA-bind_4 (Myb_DNA-bind_4), APETALA2 (AP2), and Fantastic Four meristem regulator (FAF). Further functional enrichment analysis showed that 48 terms related to abiotic stress regulation and floral development were significantly enriched in ten species, whereas no significantly enriched terms were found in four species. Interestingly, the largest number of enriched terms was detected in *Citrus sinensis* (L.) Osbeck, accounting for 54.17% of all significantly enriched functional terms. Finally, we analyzed AP2 and trihelix gene families (Myb_DNA-bind_4) due to their significant enrichment in SSR-containing genes. The results indicated that whole-genome duplication (WGD) and whole genome triplication (WGT) might have played major roles in the expansion of the AP2 gene family but only slightly affected the expansion of the trihelix gene family during evolution. In conclusion, the identification and comprehensive characterization of SSR markers will greatly facilitate future comparative genomics and functional genomics studies.

## INTRODUCTION

Generally, forest and fruit trees undergo a long juvenile period before flowering or fruiting, making the tree breeding process extremely long and challenging. An important prerequisite for plant breeding is the understanding of genetic variation. Molecular markers are useful tools for plant improvement as they can detect existing mutations in the genome and decode the genetic control of important traits, such as disease resistance, abiotic stress tolerance, and fruit quality attributes, to shorten the time required for obtaining new varieties with superior quality.

Molecular markers are nucleotide sequences that can reveal the distribution of genes and the expression of phenotypic traits among individuals by analyzing DNA fragments that encompass different genetic information^[1]^. Based on the detection method, molecular markers can be divided into three classes: hybrid-based markers, such as restriction fragment length polymorphisms (RFLPs); PCR-based markers, such as random amplified polymorphic DNA (RAPD) markers, simple sequence repeats (SSRs), and amplified fragment length polymorphism (AFLP) markers; and DNA sequence-based markers, such as single nucleotide polymorphisms (SNPs)^[2, 3]^. Among these molecular markers, SSR markers or microsatellites, are one of the most commonly used markers in plant breeding, gene flow analyses and genetic diversity assessments^[4]^. According to the arrangement of nucleotide(s) in the repeat unit, SSRs are classified as perfect, imperfect, and compound microsatellites^[5]^. Perfect SSRs are defined as continuous repetitions without any interruption (e.g., (AG)_12_), while repeated sequences in the imperfect SSR interrupted by different bases that are not repeated (e.g., (AG)_10_TC(AG)_8_). Compound SSRs contain two adjacent distinct SSRs (e.g., (AG)_10_(TC)_8_).

SSR markers are dispersed over the coding and non-coding regions of all prokaryotic and eukaryotic genomes analyzed to date^[6]^. Analyses of the occurrence of microsatellites in some plant and animal species indicate no apparent association between genome size and SSR density^[7, 8]^. SSR markers show high levels of variation in motif frequency and microsatellite class^[9]^. Generally, coding sequences exhibit a relatively low SSR density, as a high mutation rate of SSRs may affect gene function^[10, 11]^. SSRs in coding regions are mostly tri- and hexa-nucleotide SSRs and are assumed not to cause frame shift mutations, as they are multiples of three nucleotides^[12, 13]^. Emerging evidence suggests that SSRs may regulate gene transcription, translation, DNA methylation, mRNA stability, chromatin structure, and metabolic activities^[14−16]^.

With the rapid development of next-generation sequencing (NGS), it is now possible to screen SSR markers in different species in a more efficient and cost-effective way^[17−19]^. In fact, SSR markers have been developed based on the genome sequences of several trees such as *C. sinensis*, *Citrus maxima *Merr., *Jatropha curcas *L., and *Salix brachycarpa *Nutt.^[18, 20−23]^. In the present study, we systematically analyzed and compared the characteristics of SSR markers based on the released genome sequences of 14 trees, including ten eudicots, one monocot, one basal angiosperm, one gymnosperm, and one Lycopodiophyta species. Moreover, the potential functions of SSR-containing sequences were further investigated based on enriched Gene Ontology (GO) annotations. This research deepens our understanding of the characteristics of SSRs and their potential biological functions in trees, thereby providing new information for future breeding programs.

## RESULTS

### Comprehensive SSR identification

SSR identification was performed in 14 forest and fruit trees, including ten eudicots, one monocot (*Elaeis* *guineensis *Jacq.), one basal angiosperm (*Amborella* *trichopoda *Baill.), one gymnosperm (*Picea abies *(L.) H. Karst.), and one Lycopodiophyta (*Selaginella* *moellendorffii *Hieron.) species. Many of the ten eudicot species were fruit trees, including *Prunus persica* (L.) Batsch,* Vitis vinifera* (L.), *C. sinensis*, *Theobroma cacao* (L.), *Coffea canephora *Pierre ex A.Froehner, and *Carica papaya *(L.). We also selected some representative tree species, including *Populus* *trichocarpa *Torr. & A.Gray ex. Hook., *Eucalyptus* *grandis *W.Hill, *Salix purpurea *(L.), and *J.* *curcas*.

**Figure 1:**
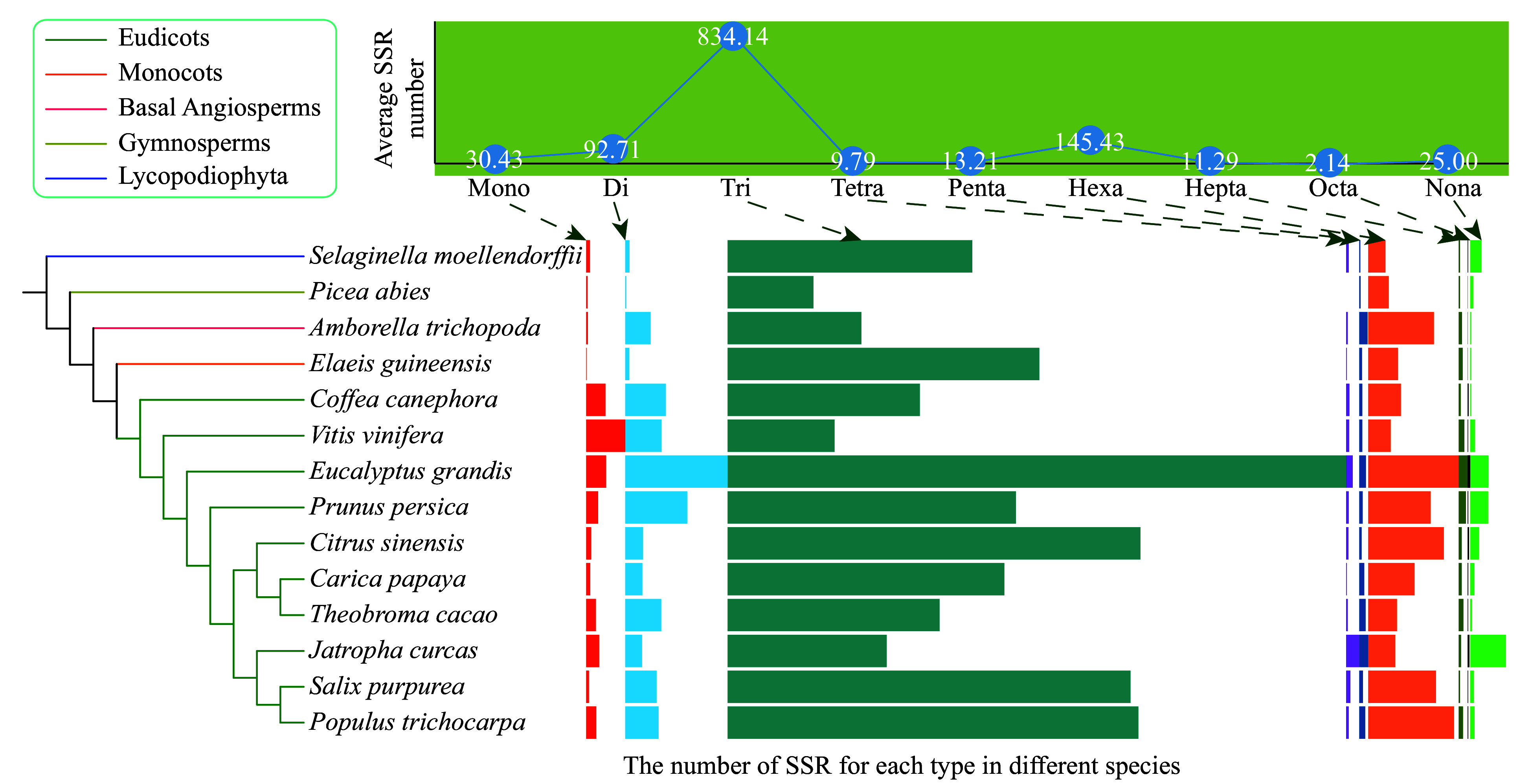
The average number of nine types (mono- to nona-) of simple sequence repeats (SSRs) and their distribution in the 14 species, including ten eudicots, one monocot, one basal angiosperm, one gymnosperm, and one Lycopodiophyta species. The boxplots indicate the number of SSR for each type in different species. The boxplots indicate the number of SSR for each type in different species.

A total of 16,298 SSRs of mono- to nona-nucleotide repeat types were identified from 429,449 genes in the 14 species (Fig. 1, Supplemental Tables S1−S2). Most SSRs were tri-nucleotide SSRs, with an average number of ~834 per species (Fig. 1, Supplemental Table S1). This might have been because tri-nucleotide SSRs do not cause frame shifts in the coding sequences. The hexa-nucleotide SSRs ranked second among all types of SSRs in nine species, and di-nucleotide SSRs ranked second among all types of SSRs in three species (Supplemental Table S1).

### Comparative analysis of the SSRs among 14 species

**Figure 2:**
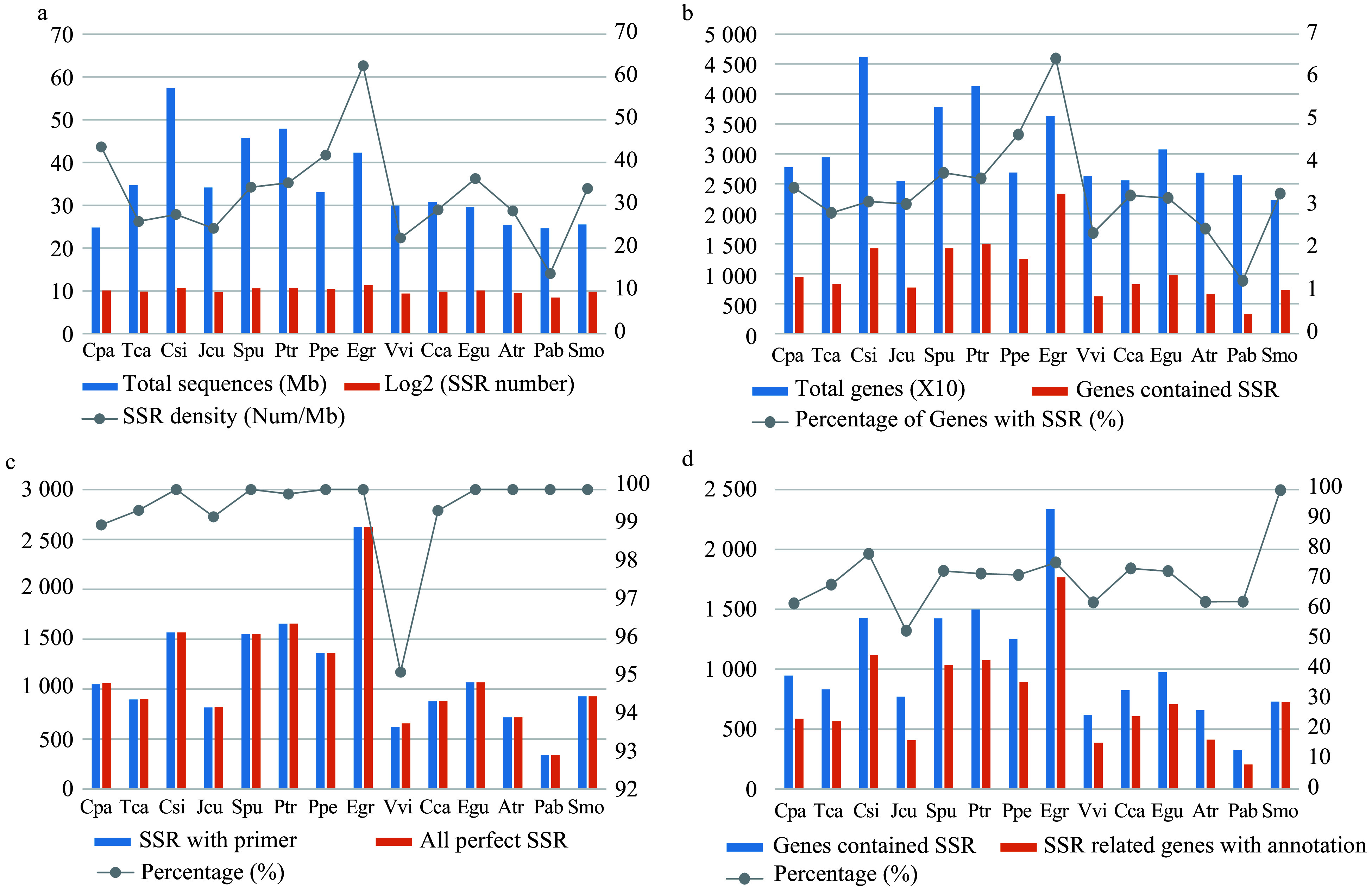
Comparison of the characteristics of simple sequence repeats (SSRs) among the 14 species. (a) The total length of genome sequences and the number and density of SSRs in each species. (b) The total number of genes and the number and percentage of SSR-containing genes in each species. (c) The total number of perfect (non-compounds) SSRs and the number and percentage of SSRs with successfully designed primers in each species. (d) The number of SSR-containing genes and the number and percentage of SSR-containing genes with Pfam annotations in each species.

*E. grandis* contained the highest number of SSRs in genes (2,647) among all 14 species, followed by *P. trichocarpa* (1,689) and *C. sinensis *(1,601) (Fig. 2a, Supplemental Table S2). By contrast, only 346 SSRs were identified in the gymnosperm species *P. abies. *Consistent with these results,* E. grandis *exhibited the highest SSR density (~63/Mb), whereas that of *P. abies *was only ~14/Mb (Supplemental Table S2).

*C.* *sinensis* had the highest number of genes (46,147) among all 14 species, whereas *E*. *grandis* had the highest number of SSR-containing genes (2,336) (Fig. 2b, Supplemental Table S2). We successfully designed primers for 100% of the identified SSRs in eight of the 14 species, whereas only 95.12% SSRs in *V. vinifera* had successful primers (Fig. 2c, Supplemental Table S2). On average, primers were successfully designed for 99.44% of the SSRs from all 14 species (Supplemental Dataset 1).

To further investigate the function of SSRs, we conducted functional annotation using the Pfam database^[24]^. *E. grandis *had the highest number of annotated SSR-containing genes (1,766), followed by *C. sinensis *(1,118) and *P. trichocarpa *(1,077) (Fig. 2d, Supplemental Table S3). This seems to be the result of high correlation between total genes and genes with annotation (*r *= 0.96)*. S*. *moellendorffii *had the highest percentage of annotated SSR-containing genes (99.73%) among all 14 species.

### Functional enrichment analysis of SSR-containing genes

**Figure 3:**
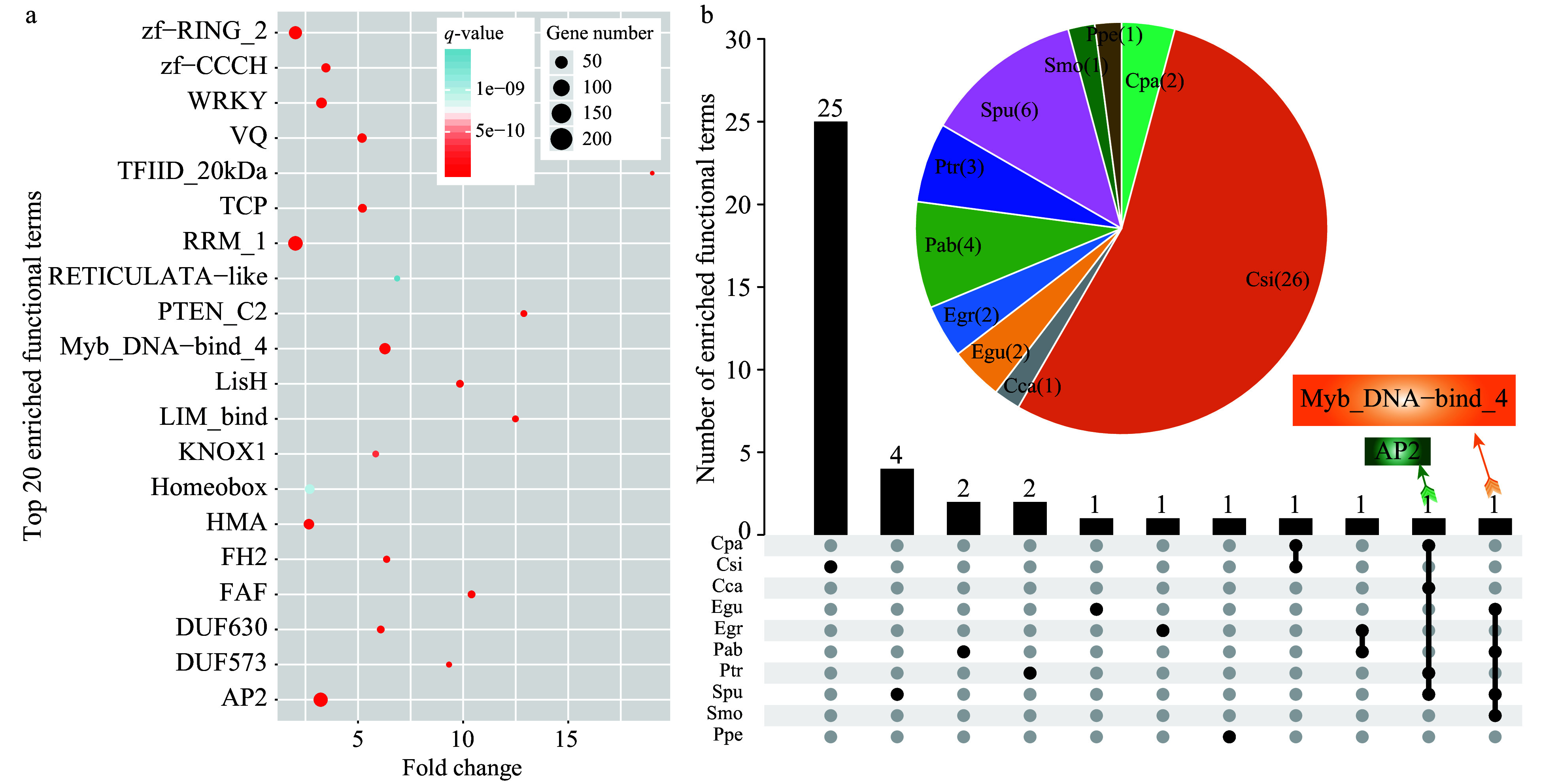
Functional enrichment analysis of SSR-containing genes in the 14 species. (a) The top 20 enriched terms based on Pfam annotations (*q*-value < 0.01, fold-change ≥ 2). The size of dots indicates the number of enriched genes in each related pathway and the color of dots represents *q*-values. (b) A Venn diagram showing enriched functional terms common or specific to each species based on Pfam annotations. The pie chart indicates enriched functional terms in each species.

A total of 10,496 annotated SSR-containing genes were identified from all 14 species, with an average annotation rate of > 70% (Supplemental Table S3). We further performed functional enrichment analysis of all SSR-containing genes and identified 50 enriched terms with a *q*-value < 0.01 and fold-change ≥ 2 (Supplemental Table S4). The fold-change indicated that the percentage of terms enriched for SSR-containing genes was comparable to that of all identified genes. The most significantly enriched term was Myb_DNA-bind 4 (Trihelix gene family), followed by Apetala 2 (AP2), fantastic four meristem regulator (FAF), and VQ motifs (Fig. 3a, Supplemental Table S4). Among the 50 enriched terms, TFIID_20kDa had the greatest fold-change of > 19-fold, followed by DUF2052, and PTEN_C2. Interestingly, we found that the most significantly enriched functional terms belonged to transcription factor families associating with the regulation of abiotic stress, and they included Myb, AP2, TCP, and WRKY family members. These results indicate that SSRs may play critical roles in stress responses in plants.

We also conducted functional enrichment analysis on SSR-containing genes in each species. The results showed that 48 terms were significantly enriched in ten species, among which the largest number of enriched terms was detected in *C. sinensis* (26 terms), accounting for 54.17% of all significantly enriched functional terms. By contrast, no significantly enriched terms were detected in four species, including *T. cacao*, *J. curcas*, *V. vinifera*, and* A. trichopoda *(Fig. 3b, Supplemental Table S5).

As shown by the Venn diagram, 25, four, two, two, one, one, and one enriched functional terms were specific to *C. sinensis*,* S. purpurea*,* P. abies*, *P. trichocarpa*, *E. guineensis*, *E. grandis*, and *P. persica*, respectively (Fig. 3b). The enriched functional term AP2 was shared by four species, including* C. papaya*, *C. canephora*, *S. purpurea*, and* P. trichocarpa*. Similarly, Myb_DNA-bind 4 was also enriched in four species, including *E. guineensis*, *P. abies*,* S. purpurea*, and* S*. *moellendorffii.* These results indicated that genes containing AP2 and Myb_DNA-bind 4 domains (a conservative domain of the trihelix family) might play important roles mediated by SSRs in these species^[25]^.

### Analysis of the AP2 gene family

#### identification and comparative analysis of the AP2 gene family

The above analyses showed that AP2 family genes were significantly enriched for SSRs; therefore, we further conducted phylogenetic and comparative analyses of this gene family. AP2 family genes contain two AP2 domains and belong to the APETALA2/Ethylene-Responsive Factor (AP2/ERF) superfamily^[26, 27]^. They play key roles in the development of reproductive and vegetative organs, such as the specification of floral organ identities, the regulation of flowering time, and the modulation of seed development^[28, 29]^.

A total of 1,649 AP2 family genes were identified from the genomes of 14 species according to the Pfam annotation (Supplemental Table S6). *S*. *purpurea* had the largest number of AP2 family genes (222), followed by *P*. *trichocarpa* (210) and *C*. *sinensis* (146). However, only 25 AP2 family genes were identified in *J. curcas*, and this number was the smallest among all 14 species. A total of 190 SSR-containing AP2 family genes were identified in the 14 species, accounting for 12.12% of all AP2 family genes. The ratio of SSR-containing AP2 genes was highest in *C*. *papaya* (20.21%), followed by* J. curcas* (20.00%) and *E. guineensis* (14.89%).

#### Gene duplication and loss of the AP2 family

**Figure 4:**
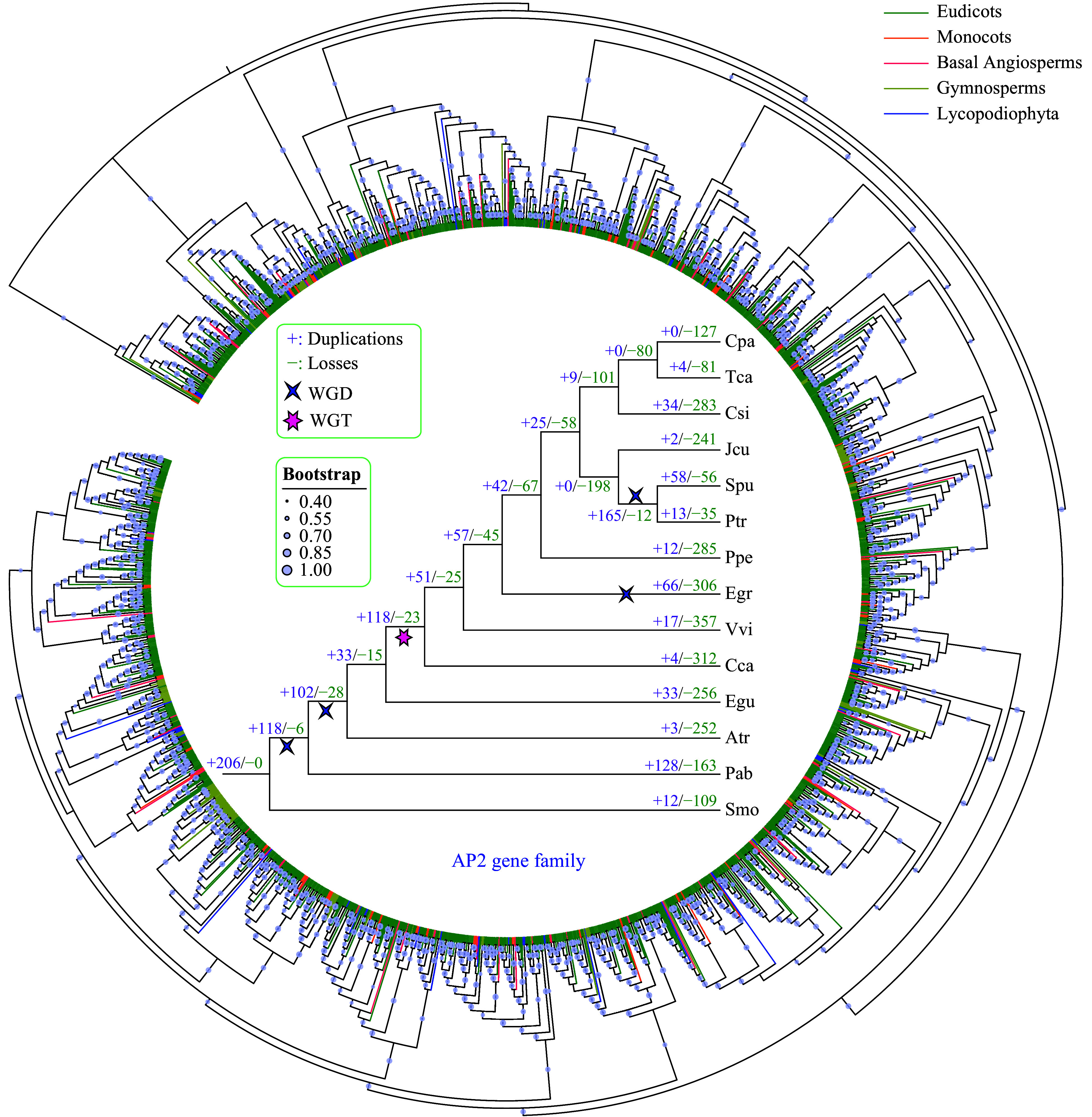
Phylogenetic and gene duplication and loss analyses of the AP2 gene family. The maximum-likelihood (ML) tree was generated based on the amino acid sequences of the AP2 gene family. The phylogenetic tree of the 14 species was constructed using FastTree software with 1000 bootstrap repeats. Bootstrap values greater than 40% are shown on each branch and are proportional to the sizes of dots. Analyses of gene duplication and gene loss events in the AP2 gene family were performed using Notung software. Gene duplication and loss events are indicated by "+" and "−", respectively, on each branch with numbers indicated. WGD and WGT events are also indicated.

To explore the evolutionary history of the *AP2* gene family, we constructed a phylogenetic tree using the amino acid sequences of 1,649 *AP2* genes from 14 species (Fig. 4, Supplemental Fig. S1). According to the topology of the phylogenetic tree, genes from different *AP2* gene families were clustered into different groups. We then further analyzed the duplication and the loss of *AP2* family genes in the 14 species using Notung software, through the reconciliation between species tree and the AP2 phylogenetic tree.

Our results indicate that the AP2 family has undergone more gene duplication than gene loss events in almost all examined species, except for *S. purpurea *(Fig. 4). *P*. *abies* had the most gene duplication events (128), whereas no AP2 gene duplication was detected in *C. papaya*. *C. canephora* had the largest number of gene loss events (312), followed by *E.* *grandis* (306) and *P.* *persica* (285). Furthermore, we found that whole-genome duplication (WGD) and whole-genome triplication (WGT) played a major role in the expansion of the AP2 gene family during evolution. For example, more gene duplication than gene loss events occurred in four of the five WGD or WGT events based on the phylogenetic tree of the 14 species.

### Analysis of the *Trihelix* gene family

#### identification and comparative analysis of the trihelix gene family

We found that the trihelix family was also significantly enriched for SSR-containing genes; therefore, we conducted comparative analyses of these genes among all 14 species. Trihelix transcription factors play important roles in a series of developmental processes, including the morphogenesis of various floral organs, leaves and trichomes, embryonic development, the regulation of light-dependent gene expression, and responses to multiple biotic and abiotic stresses.

Based on the Pfam annotation, we identified 471 trihelix genes from the 14 genomes (Supplemental Table S7). *P. trichocarpa *had the largest trihelix family (58), followed by* S. purpurea* (55) and *C. sinensis* (48). By contrast, only ten trihelix family genes were identified in *J. curcas*. Among all identified trihelix family genes in the 14 species, 104 contained SSRs. *S. moellendorffii* and *J. curcas* had the highest percentage of SSR-containing trihelix family genes (40% in both species), whereas *T. cacao* and *C. sinensis* had only 9.38% and 14.58% SSR-containing trihelix genes, respectively.

#### Gene duplication and loss of the trihelix gene family

**Figure 5:**
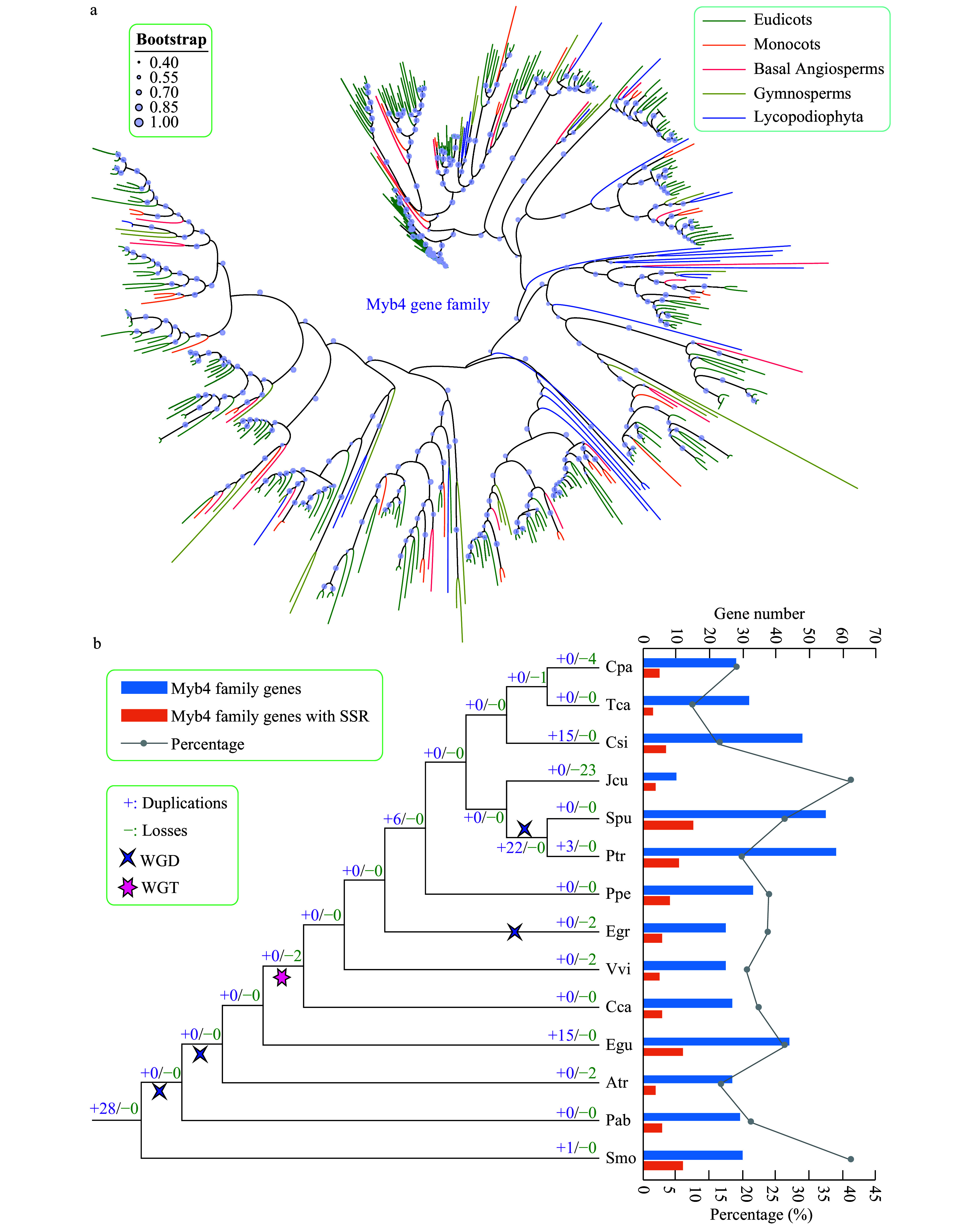
Phylogenetic and gene duplication and loss analyses of the trihelix gene family. (a) The maximum-likelihood (ML) tree was generated based on the amino acid sequences of the Myb_DNA-bind_4 gene family. The phylogenetic tree of the 14 species was constructed using FastTree software with 1,000 bootstrap repeats. Bootstrap values above 40% are shown on each branch and are proportional to the sizes of dots. (b) Analysis of gene duplication and gene loss events of the Myb_DNA-bind_4 gene family was performed using Notung software. Gene duplication and gene loss events are denoted by "+" and "−", respectively, on each branch with numbers indicated. The blue and orange bars indicate the number of trihelix family genes and SSR-containing trihelix family genes in each species, respectively. The line chart shows the percentages of SSR-containing trihelix genes in each species.

We constructed a phylogenetic tree using the amino acid sequences of trihelix genes from 14 species to further explore their evolutionary characteristics (Fig. 5, Supplemental Fig. S2). Gene duplication and gene loss events of the trihelix family were analyzed based on the reconciliation between species and the phylogenetic tree.

The trends of gene duplications and gene losses of the trihelix family differed from those of the AP2 gene family. For example, relatively fewer gene duplication and gene loss events were observed in almost all species (Fig. 5). In general, *C. sinensis* (15) and *E. guineensis *(15) exhibited more gene duplications and gene losses compared with other species. *J. curcas* underwent more gene losses (23) than other species. Unlike the AP2 gene family, WGD and WGT only slightly influenced the expansion of the trihelix gene family during evolution, and apparent gene expansion (22) was only observed in the lineages of the common ancestor of *P. trichocarpa* and *S. purpurea*, which might have been the result of a WGD event.

## DISCUSSION

In this study, we developed SSR markers from all genes of whole-genomes in 14 tree species. Unlike crops, the breeding of fruit and forest trees is still in its infancy due to their long breeding cycles, complex genetic structures, and lack of genomic and functional information. In general, an improvement in breeding precision and a shortening of the breeding cycle are two major challenges for the genetic improvement of perennial woody plants. The selection of new varieties by traditional breeding methods is insufficient. With advancements in molecular biology, SSR markers provide a powerful means for directional genetic manipulation and improvement of tree species^[30−32]^.

Molecular marker assisted selection (MAS) combines modern molecular biology and traditional breeding. SSR markers can be used to select breeding materials at the DNA level to improve yield, quality, and resistance in tree species. The rapid development of high-throughput sequencing technology, and the substantial reduction in sequencing cost has promoted the genotyping of large-scale mapping populations, allowing for the construction of high-density genetic linkage maps^[17, 18]^. Furthermore, the whole genomes of many tree species have been sequenced and released, which facilitated the identification of SSR markers for all genes of species. Developing SSR markers that closely associate with functional genes can help select individuals with a desired phenotype at an early stage of tree growth, significantly improving breeding efficiency. Here, a total of 16,298 SSRs were identified in 429,449 genes of 14 representative trees. In addition, we successfully designed primers for 16,081 SSRs. Therefore, these resources will promote molecular marker assisted selection applied in tree breeding.

Furthermore, several significantly enriched terms were detected in SSR-containing genes, and most enriched functional terms were transcription factors (TFs). Transcription regulation of gene expression plays important roles in many biological processes such as cell morphogenesis, signal transduction, and responses to environmental stress^[33−35]^. Plant growth and productivity are greatly threatened by environmental biotic and abiotic factors^[34, 36]^. To adapt to environmental changes, plants have evolved a large number of TFs to combat adverse effects^[37−39]^. In this study, SSR markers were found to be significantly enriched in TFs associated with abiotic stress and floral development, and they included members of the Myb_DNA-bind 4, AP2, TCP, and WRKY families. The large number of TF families enriched for SSRs might have been a result of the various environmental conditions these species live in, causing the evolution of different TF families to cope with different adverse environmental factors.

The Myb_DNA-bind 4 was also known as the trihelix gene family^[25]^. The trihelix proteins were one of the earliest TF families found in plants but have attracted attention only recently^[40, 41]^. They were first classified as GT factors and further classified into five clades, GT-1, GT-2, SH4, GTg, and SIP1^[40]^. Among these 14 species, the trihelix gene family was only reported in *P. trichocarpa* at the whole-genome level ^[42]^. Here, we detected 471 trihelix family genes from the whole-genome of 14 trees, ranging from 10 (*J. curcas*) to 58 (*P. trichocarpa*). Until now, the function of trihelix family genes has been investigated only in some model plants or major crops, such as Arabidopsis, tomato, rice, soybean, and wheat^[43−49]^. Besides a role in regulating the development of flowers, stomata, embryos, and seeds, recent studies have found that some members of the trihelix gene family can respond to biotic and abiotic stresses such as disease, salt, drought, and cold stress^[40]^. Among forest trees, there are only a few studies on trihelix family gene function in *P. trichocarpa*, while it was rarely reported in other species^[42]^. In *P. trichocarpa*, some trihelix genes are responsive to osmotic stress and can be induced by phytohormones, including abscisic acid, salicylic acid, and methyl jasmonate^[42]^. Moreover, the inhibition of *PtrGT10* expression enhances the scavenging ability of reactive oxygen species and reduces cell death^[42]^. Here, we conducted systematic comparative analyses and phylogenetic analyses for these family genes in *P. trichocarpa* and other trees. Based on these analyses, we easily obtained the homologous genes between *P. trichocarpa* and each of other examined trees. Therefore, the previous studies of *P. trichocarpa* genes provide a good reference for the functional studies of homologous trihelix family genes in other trees. The functions of trihelix genes are becoming clear with the identification and characterization of more members in this family. Therefore, our study provides rich gene resources for the functional research of trihelix family genes in trees. Especially, trihelix family genes that contain SSRs were significantly enriched in four species, including *E. guineensis*, *P. abies*, *S. purpurea*, and *S. moellendorffii*. However, there are few reports on this gene family in these four species. Therefore, this study lays the foundation for future studies on the function of trihelix family genes in these species.

SSR markers were also significantly enriched in AP2 family genes that are associated with abiotic stress responses. Members of the AP2 gene family have different functions in regulating plant development and various stress responses^[50, 51]^. The AP2 family contains one or two tandem AP2 domains, which shows a relatively high similarity between different genes^[52, 53]^. Members of the AP2 family are expressed primarily in young organs and function as key regulators of plant growth and development, including floral meristem establishment, floral organ identity specification, the regulation of floral homeotic gene expression, and the regulation of ovule development^[54−56]^. Among 14 tree species, the AP2 gene family has been reported in *V. vinifera*, *P. persica*, and *P. trichocarpa *at the whole-genome level^[57−59]^. Here, we detected 1,649 AP2 family genes from the whole-genome of 14 trees, ranging from 25 (*J. curcas*) to 222 (*S. purpurea*). Our results show that AP2 family genes that contain SSRs were significantly enriched in four species, including *C. papaya*, *C. canephora*, *P. trichocarpa*, and *S. purpurea*. A previous study implied that AP2 family genes play important roles in fruit growth and development in peach^[58]^. Furthermore, we conducted a phylogenetic reconstruction using all AP2 family genes of 14 species, which provides good guidance for functional studies of AP2 family genes based on the homologous relationship with model species. Generally, whole genome duplications (WGD) or triplications (WGT) are the major sources for the diversification and specification of gene function^[60−62]^. Our results suggest that WGD and WGT events may play important roles in the expansion of the AP2 gene family, which further contributed to the evolution of these family genes. Therefore, these findings also provide new insights into the evolution and functions of SSR-containing genes for future comparative and functional genomics studies of these species and other related tree species.

## CONCLUSION

In summary, we identified SSRs from whole-genomes of 14 tree species and investigated the characteristics of these SSRs in major forest and fruit trees through comparative analysis. A total of 50 significantly enriched terms were detected by comparing all identified SSR-containing genes with genes that have Pfam annotations. Most enriched functional terms were TFs that related to abiotic stress regulation, and they included Myb, AP2, TCP, and WRKY gene families. Furthermore, enriched functional terms that were common or specific to each species were analyzed, and seven species were enriched for specific functional terms. Finally, we found that functional terms AP2 and Myb_DNA-bind 4 were each significantly enriched in four species. Taken together, our findings provide valuable insights into the evolution and functions of SSR-containing genes for future functional genomics studies and valuable genetic resources for developing markers for the breeding of these tree species.

## Materials and Methods

### Collection of public data

The protein sequences and coding sequences of each species were obtained from Phytozome (https://phytozome.jgi.doe.gov/pz/portal.html), ensemble (http://useast.ensembl.org/index.html), NCBI (https://www.ncbi.nlm.nih.gov) and other related databases (Supplemental Table S1). Coding sequences with alternative splicing were removed using Perl script, and only non-redundant sequences were used for analysis.

### Identification of SSRs

All SSRs were identified from the above-mentioned coding sequences in the 14 species using the Microsatellite identification tool (MISA)^[63]^ with the following parameters: monomers (≥ 16×), 2-mers (≥ 8×), 3-mers (≥ 6×), 4-mers (≥ 5×), 5-mers (≥ 4×), 6-mers (≥ 4×), 7-mers (≥ 3×), 8-mers (≥ 3×), and 9-mers (≥ 3×) according to a previous report^[64]^. The maximum distance between two SSRs in a compound sequence was less than 100 bp.

### Primer design for the SSRs

Primers were designed for all identified SSRs using Primer3^[65]^ as reported in a previous study^[64]^. The melting temperature (T_m_) of primers ranged between 55 and 65 °C, with an optimum T_m_ of 60 °C. The size of the primers was set to 20 nt and ranged between 18 and 27 nt. The size of the PCR products was set to 150 bp and ranged between 100 and 280 bp.

### Functional annotation and enrichment analysis

Functional annotation of SSR-containing genes and all genes was performed using the Pfam database (http://xfam.org)^[24]^. The enrichment analysis was then conducted by the comparison of SSR-containing genes in related pfam term, SSR-containing genes with pfam annotation, all genes in related pfam term, and all genes with pfam annotation in each species. Enrichment analysis was conducted using the scipy package of Python^[66]^. R was used to perform Bonferroni correction on the *p*-values obtained by significance analysis. Parameters used to define significant enrichment terms were *q*-value < 0.01 and fold-change ≥ 2. A Venn diagram of enriched terms specific to, or shared by the species, was generated using TBtools program^[67]^.

### Identification of transcription factor gene families

Domains were predicted based on the protein sequences of each species by searching the Pfam database^[68]^. Proteins containing "AP2" (PF00847) and "Myb_DNA-bind_4" (PF13837) domains were extracted using a customized Perl program with an e-value < 1e-4. The Conserved Domains Database (CDD) and the Simple Modular Architecture Research Tool (SMART) were used to validate the domains and ensure accuracy according to previous reports^[53, 69−71]^.

### Inference of gene duplication and gene loss

The protein sequences of AP2 and trihelix family members were aligned using Mafft software (v7.471) with maxiterate 1,000^[72]^. A phylogenetic tree was constructed using FastTree software (v2.1.11) with 1000 bootstrap replications^[73]^. The maximum likelihood method and JTT (Jones-Taylor-Thorton) model were used for phylogenetic analysis. The phylogenetic trees of AP2 and trihelix gene families were constructed using the iTOL program^[74]^. Gene duplication and gene loss events in these two gene families were analyzed using the Notung2.9 program according to previous reports^[75−77]^.

## SUPPLEMENTARY DATA

Supplementary data to this article can be found online.
