# Supplementary figures and images for: Comprehensive identification and characterization of simple sequence repeats based on the whole-genome sequences of 14 forest and fruit trees
Source: For Res (Fayettev). 2021 Apr 21;1:7. doi: 10.48130/FR-2021-0007 (PMC11524223; doi:10.48130/FR-2021-0007)

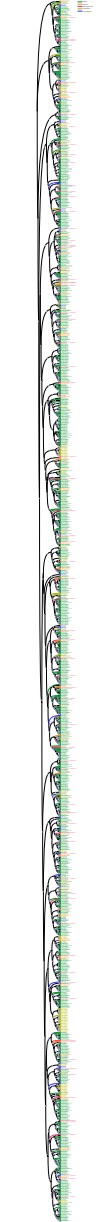

Supplement: Supplementary file 1 — Supplementary data to this article can be found online. [file FR-2021-0007-S1.zip › 10.48130_FR-2021-0007-Suppl-FigureS1.pdf]

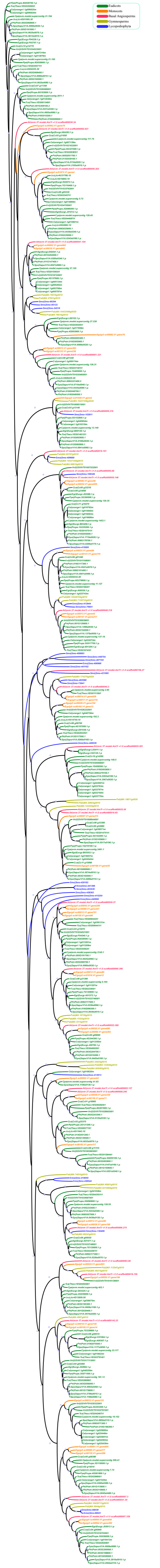

Supplement: Supplementary file 1 — Supplementary data to this article can be found online. [file FR-2021-0007-S1.zip › 10.48130_FR-2021-0007-Suppl-FigureS2.pdf]
